# Establishment of the World Health Organization First International Standard for Factor XII, Plasma, Human

**DOI:** 10.3389/fmed.2018.00046

**Published:** 2018-02-28

**Authors:** Helen V. Wilmot, Jason Hockley, Peter Rigsby, Elaine Gray

**Affiliations:** ^1^Haemostasis Section, National Institute for Biological Standards and Control, Potters Bar, United Kingdom; ^2^Biostatistics Group, National Institute for Biological Standards and Control, Potters Bar, United Kingdom

**Keywords:** International Standard for factor XII, factor XII functional activity, factor XII antigen, multicenter study, measurement of factor XII

## Abstract

Until recently, the role of factor XII (FXII) in hemostasis was not considered to be important since patients with FXII deficiency do not present with bleeding. The activation of FXII by agents including mast cells and platelet polyphosphates suggests that it may have a role in thrombogenesis. The inhibition of FXII therefore presents an option for antithrombotic therapy, and antibodies and inhibitors are already in development. Assays for FXII will be required to support these technologies, and an international standard (IS) for FXII would be useful for the development of these methods and for the clinical monitoring of patients. The purpose of this study was to develop an IS for FXII, with values for functional activity (FXII:C) and antigen (FXII:Ag). Double-spun normal plasma was pooled, filled into siliconized glass ampoules, and freeze-dried to prepare the candidate material. Data from 20 laboratories using the one-stage clotting assay were used to assign the functional activity value in units (u). The antigen value was calculated using data from eight laboratories that carried out antigen assays. Each laboratory was requested to collect two local normal plasma pools. Units of activity and antigen were calculated relative to these pools, as is usual for new coagulation factor analytes. The amount of activity or antigen in 1 ml of normal plasma from each pool was taken to be 1 unit. A total of 566 donors were used across the pools for the FXII:C study and 216 donors for the FXII:Ag study. The overall geometric mean per ampoule for FXII:C was 0.86 u and for FXII:Ag was 0.80 u. The inter-laboratory variation was 10 and 11%, respectively (expressed as the geometric coefficient of variation). Based on these data, the candidate was deemed suitable for use as an IS for FXII. In 2017, the candidate was established by the World Health Organization (WHO) Expert Committee on Biological Standardization as the WHO first IS for blood coagulation FXII, Plasma (National Institute for Biological Standards and Control code 15/180). The values assigned were 0.86 international units (IU) of functional activity (FXII:C) per ampoule and 0.80 IU/ampoule of antigen (FXII:Ag).

## Introduction

Factor XII (FXII) is an important coagulation factor *in vitro* because contact activation of FXII is required for the activated partial thromboplastin time (APTT) assay, a widely used diagnostic test. Historically, the role of FXII *in vivo* was considered less important, since FXII patients do not suffer from abnormal bleeding and, similarly, FXII-deficient mice are healthy and free from bleeding disorders (1). It was later discovered that FXII-deficient mice had defective thrombus formation (2), suggesting that FXII may have a role in thrombosis. Since then, other substances such as platelet polyphosphates (3) and neutrophil extracellular traps (4) have been shown to be *in vivo* activators of FXII. FXII has also been linked with inflammatory responses such as acute respiratory distress syndrome (5), anaphylaxis, and hereditary angioedema (6). With the knowledge that the depletion of FXII does not affect normal hemostasis, this presents opportunities for therapies targeting FXII to be designed. Various therapies are in development (7, 8), and these new technologies require supporting assays for FXII, for which there is currently no International Standard (IS). A plasma IS for FXII functional activity (FXII:C) and antigen (FXII:Ag) would support the development of such assay methods and clinical monitoring of patients.

The processing conditions required to make a standard for FXII are the same as those required for a FXI standard, since both are contact activation factors and exposure to glass or cold results in the activation to FXIIa and FXIa, respectively. The first IS for FXI has just been replaced by the second IS due to stock depletion (9), so this presented an ideal opportunity to use the same processed material to generate the first IS for FXII. This study presents the results of the collaborative study conducted to assign the material with values for functional activity (FXII:C) and antigen (FXII:Ag).

## Materials and Methods

### Candidate World Health Organization (WHO) First IS for FXII, Plasma [National Institute for Biological Standards and Control (NIBSC) Code 15/180]

The candidate material (15/180) is the same as used for the second IS for FXI, Plasma, details of which have been published elsewhere (9). Briefly, plasma that had been prepared by the centrifugation of whole blood collected into CPD (citrate-phosphate-dextrose) adenine anticoagulant was purchased from the UK Blood Service. The plasma had been subjected to a second centrifugation step to remove cellular material and rapidly frozen at −70°C. The individual donations all tested negative for HBsAg, anti-HIV-1 and HIV-2 antibodies, and anti-HCV. Immediately prior to filling, the plasma was thawed at 37°C and pooled. A concentration of 40 mM HEPES and 1% w/v glycine was used for the final formulation to improve stability (10). Care was taken to avoid the activation of FXII by cold activation or contact with glass, and the preparation was filled into siliconized glass ampoules and freeze-dried in accordance with recommendations for the preparation of international standards (11). The product had the following physical characteristics: a mean fill mass of 1.0094 g (cv = 0.3%); a mean dry weight of 0.0928 g (cv = 0.2%); a mean residual moisture of 0.605% (cv = 13.7%); and a mean oxygen head space of 0.23% (cv = 31.4%). A total of 6,000 ampoules of 15/180 were produced. The activation status of candidate 15/180 with regard to the presence of FXIIa was assessed. Using the non-activated partial thromboplastin time (NAPTT) (carried out as described (12) except that FXII-deficient plasma was used), it was found that 15/180 did not shorten NAPTT of FXII-deficient plasma. In addition, results from FXIIa (pre-kallikrein activator) assays (13) indicated undetectable levels of FXIIa. While these test data do not preclude the presence of FXIIa in 15/180, the level present would be unlikely to affect potency estimates of FXII. The long-term stability of 15/180 has been assured by accelerated degradation studies where ampoules of product stored at elevated temperatures are assayed relative to ampoules stored at ultra-low temperature (−150°C). The predicted loss of FXII:C and FXII:Ag per year when the ampoules are kept at a storage temperature of −20°C was 0.0 and 0.024%, respectively.

### Samples in the Study

The candidate material (NIBSC code 15/180) was provided to the participants as coded duplicate samples, A and B. New coagulation factor standards are assigned relative to normal plasma pools, assuming that 1 ml of plasma contains 1 unit of activity or antigen. Therefore, participants were also asked to collect fresh local normal plasma pools for use both fresh (coded P1) and subsequently frozen (coded P2) in the study, for use as the standard. NIBSC in-house studies on both FXII functional activity and FXII antigen have shown that using fresh plasma, compared to the same pool of plasma after freezing, does not produce significantly different results. Therefore, for the participants unable to collect fresh plasma pools, frozen plasma pools could be used as an alternative. Overall, the pools used to assign the functional activity contained plasma from 566 donors, and the pools used for the antigen assignment comprised 216 donors.

### Collaborative Study

There were 20 laboratories from 10 countries (Austria, Canada, Croatia, Denmark, France, Germany, Netherlands, Spain, United Kingdom, and USA) that took part in the FXII:C assignment. Of these, there were seven clinical laboratories, four therapeutics producers, six diagnostics manufacturers, two regulatory laboratories, and one research laboratory. Eight laboratories took part in the FXII:Ag assignment. These laboratories were from Canada, Denmark, France, Germany, United Kingdom, and USA, comprising one clinical laboratory, one diagnostics manufacturer, four therapeutics producers, and two regulatory laboratories.

Participants were asked to assay coded duplicates A and B against local normal plasma pools and FXII:C and/or FXII:Ag using their usual in-house methods. Laboratories were provided with a protocol for the collection of plasma pools and a separate protocol giving guidance for the randomization of samples and for sufficient replication to enable statistical analysis. Four independent assays were requested for each analyte. FXII is part of the intrinsic pathway and is measured using the APTT assay. APTT reagents contain phospholipid and an activator(s) of the contact pathway (such as ellagic acid). When used with FXII-deficient plasma, the clotting time upon the addition of calcium can be used to calculate the FXII present in a given sample, relative to a standard. All participants in the FXII:C study used one-stage clotting assays with an APTT reagent. Thirteen different APTT reagents were used across different instruments and sources of deficient plasma. The sources of APTT reagent (together with the activator and the range of activation times used) and deficient plasmas used are listed in Tables 1 and 2. Table 3 lists the type of plasma pools used in the functional and antigen assays. For the antigen assays, three different commercial kits or paired antibody sets were used, plus one in-house method (14) (Table 4).

**Table 1:** Activated partial thromboplastin time (APTT) reagents used by the participants for FXII:C assays.

APTT reagent (activator)	Number of laboratories	Range of activation times used (s)
Actin FS (ellagic acid)	4	180
Actin FSL (ellagic acid)	3	180
APTT-SP (silica)	2	300
Cephascreen (ellagic acid)	1	240
Cephen (silica)	1	240
CK Prest (kaolin)	3	240
Dapttin (silica)	1	240
DG-APTT (ellagic acid)	1	300
Pathromtin SL (silica)	3	120–180
PTT A (silica)	1	240
Siron LS (ellagic acid)	1	240
SynthAFax (ellagic acid)	2	300
SynthASil (silica)	5	180–300

**Table 2:** Sources of deficient plasma used by the participants for FXII:C assays.

Deficient plasma	Number of laboratories
Affinity Biologicals	3
Dade	1
Diagnostic Grifols	1
Hyphen BioMed	1
Instrumentation Laboratory	4
Siemens	7
Stago	5
Stago (ImmunoDeficient)	1
Technoclone	4
Werfen	1

**Table 3:** Type of plasma pool used by participants for FXII functional and FXII antigen assays.

Plasma pool	Number of laboratories in FXII:C study	Number of laboratories in FXII:Ag study
Commercial frozen	6	3
Local fresh	11	2
Local frozen	10	1
Lyophilized and frozen	1	2

**Table 4:** List of antigen kit/paired antibodies used by the participants for FXII:Ag assays.

Antibody/kit source	Number of laboratories
FXII-EIA paired antibodies, Affinity Biologicals	3
Assay Pro AssayMax	1
Cedarlane paired antibodies	3
In-house antibodies	1

Raw data from the participants were returned to NIBSC for parallel line analysis (15) with CombiStats software (CombiStats™, Version 5.0, Council of Europe). A log_10_ transformation of the assay response was used for the FXII antigen assays and for the FXII functional assays; data from nine laboratories were transformed, with the rest not requiring transformation. The mean potencies were calculated as unweighted geometric means (GMs), and the variability between assays and laboratories is shown as the geometric coefficients of variation [GCV = (10*^s^* − 1) × 100%, where *s* is the SD of the log_10_ transformed potency estimates]. In order to detect any significant outliers, the log_10_-transformed laboratory mean estimates were subjected to Grubbs’ test (16). Appropriate *t*-tests of log_10_-transformed laboratory mean estimates were used for comparisons between methods. Where a laboratory performed more than one set of assays (e.g., using a different APTT reagent or instrument), these data were treated independently (designated a, b, etc.).

This study was carried out alongside the study on the second IS for FXI, Plasma (9), and the participants’ laboratory number was assigned arbitrarily across the two studies.

## Results and Discussion

### FXII Functional Assays

**Figure 1:**
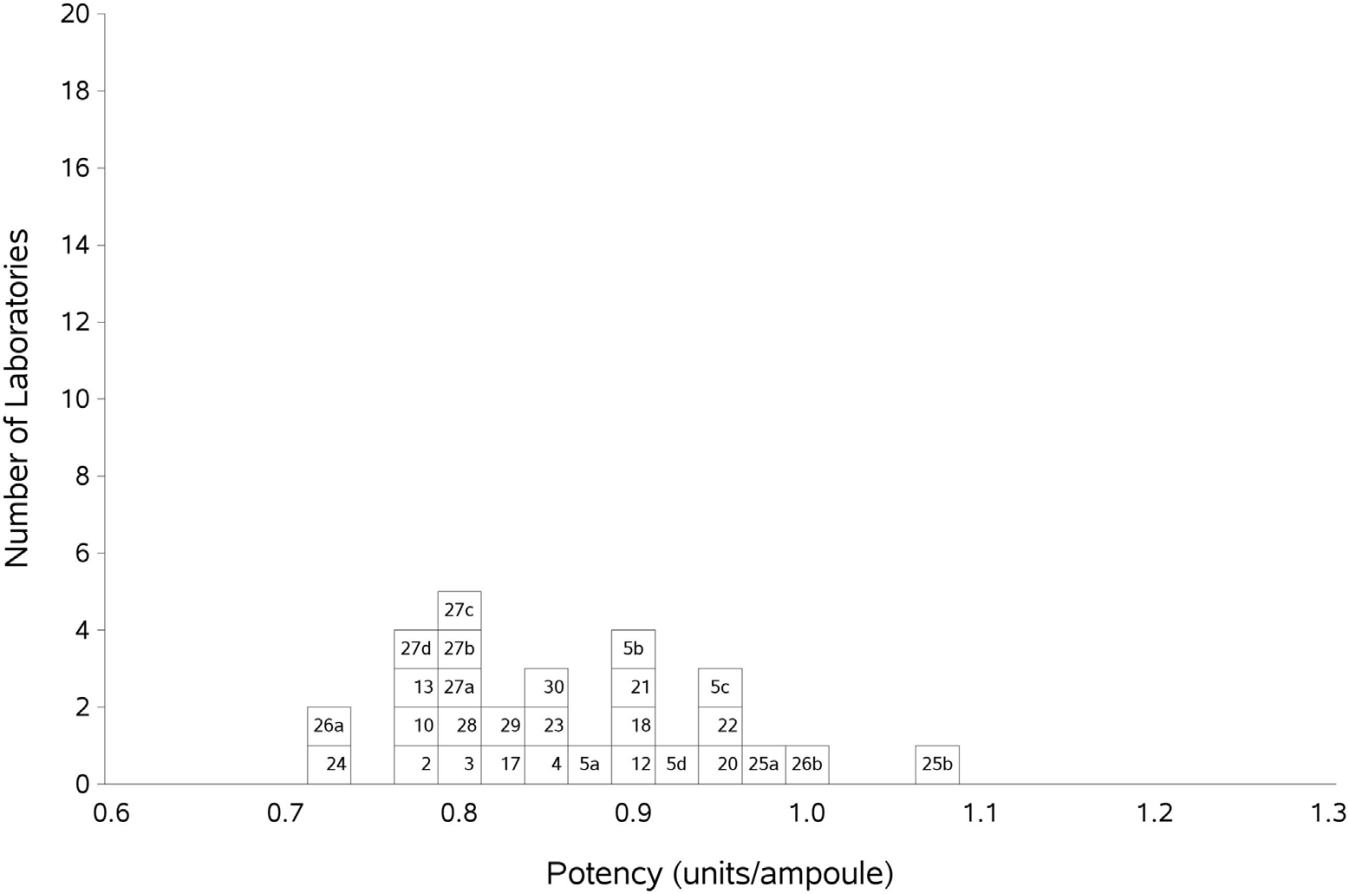
Histogram showing each laboratory’s geometric mean (GM) for FXII:C of samples A and B combined, relative to sample P, the laboratory’s local plasma pool. The overall GM was 0.86 IU/ampoule with a GCV of 10.0%.

Participants were requested to prepare two plasma pools (P1 and P2) for use as fresh in two assays and as freeze-thawed pools in a further two assays. Participants who were unable to collect fresh plasma pools were asked to use two different batches of local frozen plasma pools. Results for samples A and B, the coded duplicates, were analyzed relative to P, assuming 1 unit of FXII:C content per 1 ml of plasma pool. The GM for sample A vs P was 0.86 units per ampoule, with 95% confidence limits of 0.83–0.89. The result for sample B was 0.85 units per ampoule, with 95% confidence limits of 0.82–0.89. The intra-laboratory variation ranged from 1.7 to 20.0% for sample A and 1.9 to 19.0% for sample B, with the majority being less than 10%. Overall, the inter-laboratory GCV was 10% for each analyte. This variation is to be expected given the different plasma pools used by each laboratory. The results for A and B showed no significant difference, so the results were combined to give overall results for AB per laboratory (Table 5). The laboratory GM had a close distribution, as demonstrated in Figure 1, and showed that there was good agreement between the laboratories. The overall GM for AB vs P was 0.86 u/ampoule with an inter-laboratory GCV of 10.0%. Statistical outliers were not detected. Assays performed using fresh plasma pools were compared to those using frozen plasma pools, and there was no significant difference observed (*p* = 0.859). Of the different APTT reagents used, there was no detectable method bias.

**Table 5:** Factor XII:C, the geometric mean (GM) for each laboratory for samples A and B (coded duplicates), combined and analyzed relative to sample P, each laboratory’s local plasma pool.

Samples AB vs Sample P		

Laboratory	GM (u/amp)	GCV (%)
2	0.77	2.3
3	0.81	15.0
4	0.86	10.0
5a	0.88	11.0
5b	0.90	12.0
5c	0.94	3.8
5d	0.91	12.0
10	0.78	4.1
12	0.91	2.2
13	0.78	–
17	0.82	5.8
18	0.90	2.3
20	0.95	4.7
21	0.90	5.4
22	0.95	17.0
23	0.86	17.0
24	0.73	6.5
25a	0.97	–
25b	1.07	12.0
26a	0.72	7.9
26b	0.99	13.0
27a	0.81	6.1
27b	0.79	1.0
27c	0.79	5.5
27d	0.79	6.0
28	0.81	4.5
29	0.82	10.0
30	0.86	20.0
Overall results for AB vs P	0.86(0.82–0.89)	10.0

*Intra-laboratory variation is shown as GCV (%). The overall GM and inter-laboratory GCV for AB vs P is also shown. Figures in brackets indicate the 95% confidence limits*.

### FXII Antigen Assays

**Figure 2:**
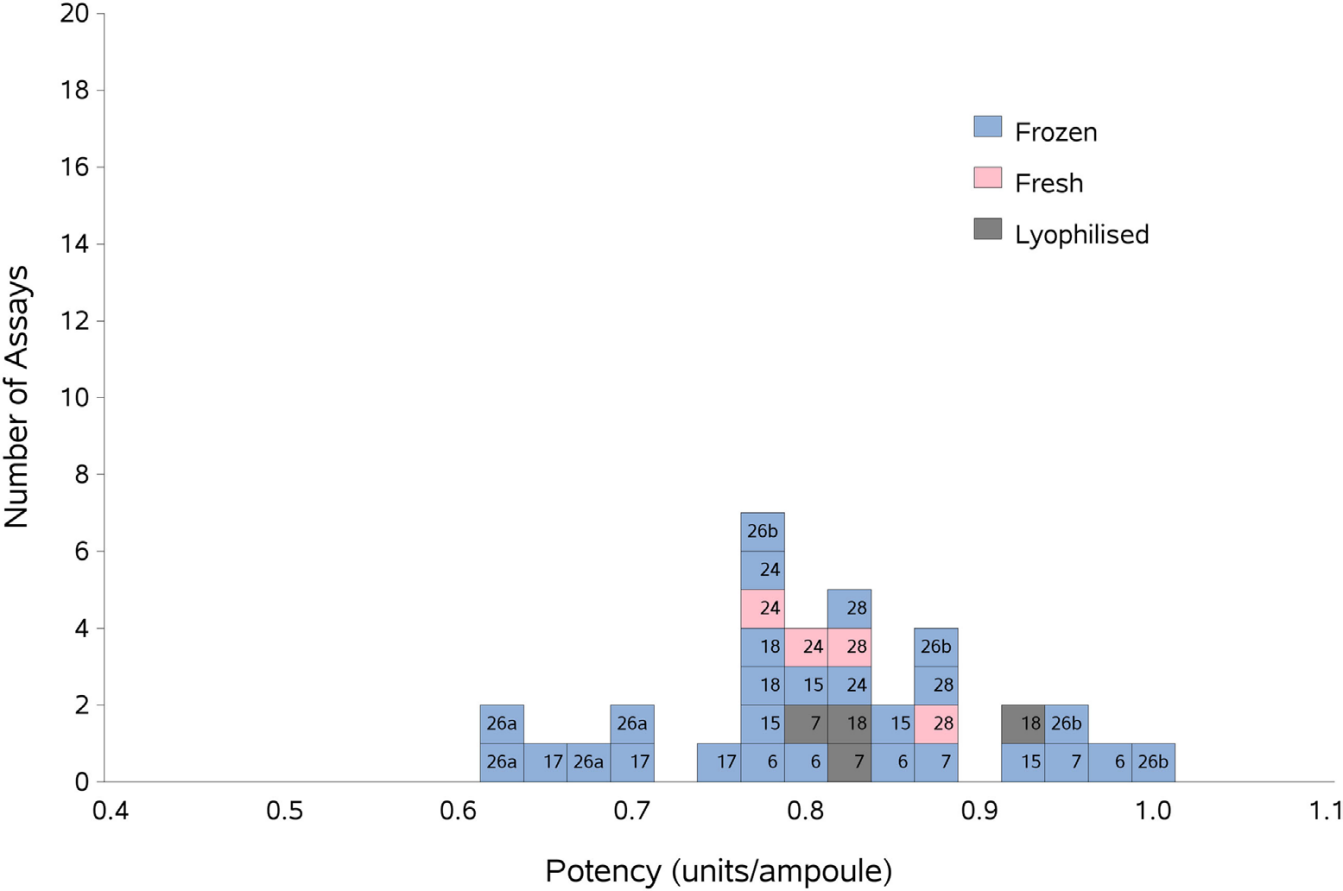
Histogram showing each laboratory’s individual assay result for FXII:Ag of samples A and B combined, relative to sample P, the laboratory’s local plasma pool. The type of pool used in each assay (fresh, frozen, or lyophilized) is also shown. The overall geometric mean was 0.80 u/ampoule with a GCV of 11.0%.

**Figure 3:**
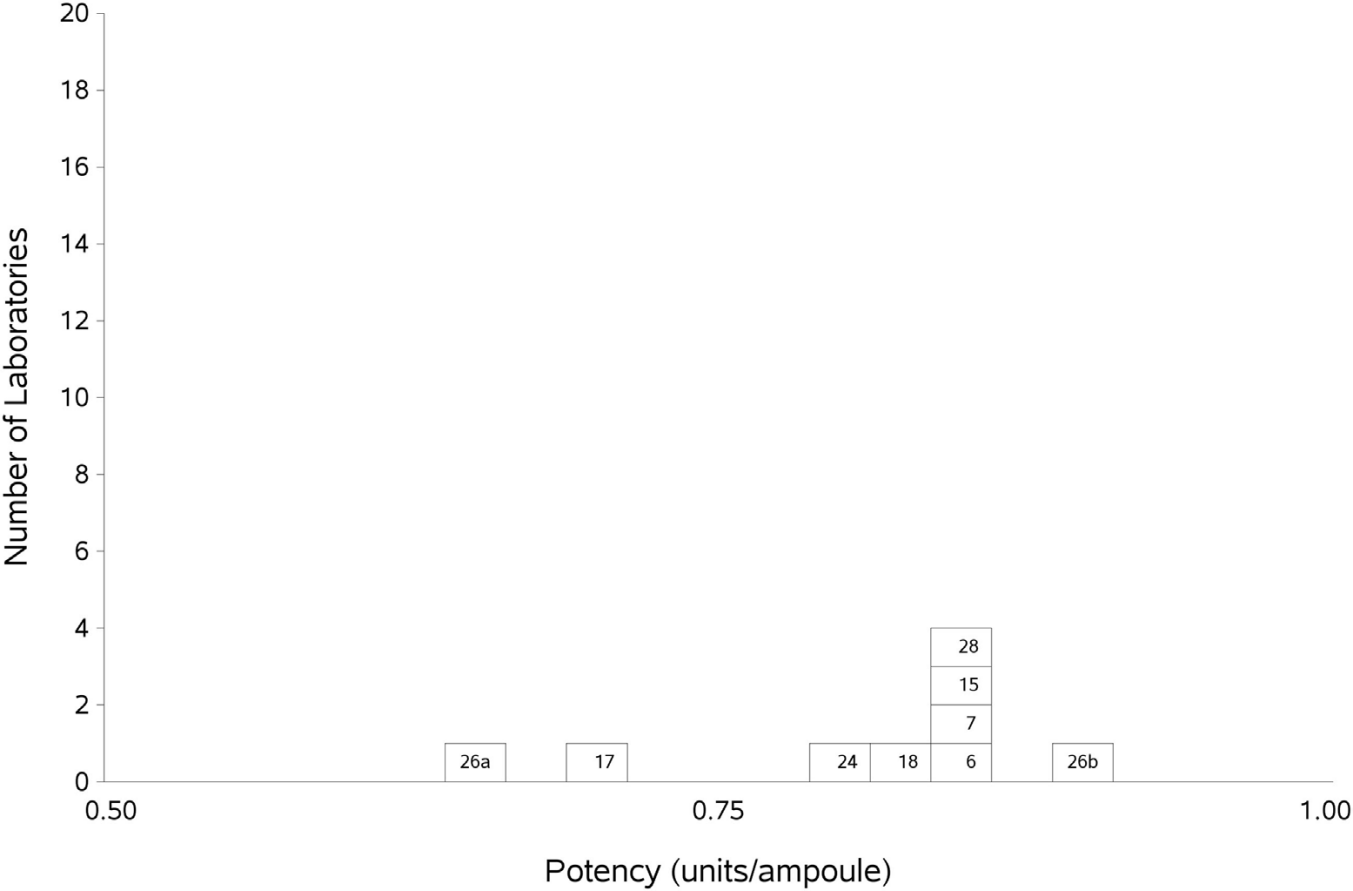
Histogram showing each laboratory’s geometric mean for FXII:Ag samples A and B combined, relative to the laboratory’s local plasma pool (sample P).

As previously, participants were requested to collect two different plasma pools to use as the standard in the assays. Results were analyzed, assuming the FXII:Ag content of 1 ml of plasma to be 1 unit. The overall GM for A against P was 0.80 u/ampoule (95% confidence limits of 0.74–0.86), compared to 0.81 u/ampoule (95% confidence limits of 0.75–0.87) for B against P. The inter-laboratory variation was 10.0 and 11.0% for A and B, respectively, showing acceptable agreement between laboratories. The results for A and B, which showed no significant differences, were combined to give a result for AB overall per laboratory (Table 6). The inter-laboratory variation was 11%, an acceptable value given the inherent variability between plasma pools. No statistical outliers were detected. Figure 2 shows each laboratory’s individual assay result, and the laboratory GMs are shown in Figure 3.

**Table 6:** Factor XII:Ag, the geometric mean (GM) for each laboratory for samples A and B (coded duplicates), combined and analyzed relative to sample P, each laboratory’s local plasma pool.

Samples AB vs Sample P		

Laboratory	GM (u/amp)	GCV (%)
6	0.84	10.0
7	0.86	7.6
15	0.84	7.6
17	0.70	7.6
18	0.82	9.3
24	0.79	3.7
26a	0.66	5.4
26b	0.90	12.0
28	0.84	3.9
Overall result for AB vs P	0.80(0.74–0.87)	11.0

*Intra-laboratory variation is shown as GCV (%). The overall GM and inter-laboratory GCV for AB vs P is also shown. Figures in brackets indicate the 95% confidence limits*.

There were not enough laboratories using fresh plasma pools to allow statistical analysis of fresh vs frozen plasma pools; however, Figure 2 shows that the results from the assays performed using fresh plasma pools (assays 1 and 3 from laboratories 24 and 28) fall in the middle of the range of results overall, suggesting that the variability of the values obtained would mostly be due to the source of the plasma pools rather than degradation due to freeze-thawing.

It is important to note that the units for activity and antigen are independent of one another and were value-assigned relative to different plasma pools. The data from the study support the value assignment of 0.86 units per ampoule for FXII:C and 0.80 units per ampoule for FXII:Ag.

## Conclusion

The results of this study were presented to the participants and experts nominated by the Scientific and Standardization Committee (SSC) of the International Society on Thrombosis and Haemostasis (ISTH). Both recommended that the candidate 15/180 (the second IS for FXI, Plasma) be used as an IS for FXII:C and FXII:Ag. In October 2017, the WHO ECBS established 15/180 as the first IS for FXII: plasma, human, with assigned values of 0.86 international unit (IU)/amp for FXII functional activity (FXII:C) and 0.80 IU/amp for FXII antigen (FXII:Ag).

## Author Contributions

HW, JH, PR, and EG designed the study. HW and EG organized the study and wrote the manuscript. JH and PR analyzed the data.

## Conflict of Interest Statement

The authors declare that the research was conducted in the absence of any commercial or financial relationships that could be construed as a potential conflict of interest.
